# Personal Strengths and Health Related Quality of Life in Dementia Caregivers from Latin America

**DOI:** 10.1155/2015/507196

**Published:** 2015-06-16

**Authors:** Stephen K. Trapp, Paul B. Perrin, Richa Aggarwal, Silvina Victoria Peralta, Miriam E. Stolfi, Eliana Morelli, Leticia Aracely Peña Obeso, Juan Carlos Arango-Lasprilla

**Affiliations:** ^1^Department of Psychology, Virginia Commonwealth University, Richmond, VA, USA; ^2^Department of Psychology, Drexel University, Philadelphia, PA, USA; ^3^Instituto San Lucas, Santa Fe, Argentina; ^4^CETYS University, Mexicali, Mexico; ^5^IKERBASQUE, Basque Foundation for Science, University of Deusto, Bilbao, Spain

## Abstract

The research literature has begun to demonstrate associations between personal strengths and enhanced psychosocial functioning of dementia caregivers, but these relationships have not been examined in the context of dementia caregivers in Latin America. The present study examined whether personal strengths, including resilience, optimism, and sense of coherence, were associated with mental and physical health related quality of life (HRQOL) in 130 dementia caregivers in Mexico and Argentina. Structural equation modeling found that the personal strengths collectively accounted for 58.4% of the variance in caregiver mental HRQOL, and resilience, sense of coherence, and optimism each had unique effects. In comparison, the personal strengths together accounted for 8.9% of the variance in caregiver physical HRQOL, and only sense of coherence yielded a unique effect. These results underscore the need to construct and disseminate empirically supported interventions based in part on important personal strengths, particularly sense of coherence, for this underrepresented group.

## 1. Introduction

Dementia is a progressive chronic illness that causes changes in the brain resulting in global cognitive and intellectual decline, memory deficits, and significant impairments in daily functioning [1]. An estimated 35.6 million people, primarily those 65 years and older, are currently living with dementia [2], with global rates expected to exceed 115.4 million by 2050 [3, 4]. The global costs of dementia care in 2010 were $604 million and will rise substantially in accordance with the increasing projected number of diagnoses [4]. The later stage dementia often requires inpatient care [5, 6], but it is common for an individual with dementia to require some degree of caregiving over the course of the disease. The majority of older individuals with dementia receive assistance from their spouse or adult children [7, 8]. In the United States alone, 15 million informal caregivers (e.g., family members and friends) provide care for individuals with dementia, and over 50% of these are residential caregivers living in the same household as the patient [3]. Although caregiving is sometimes divided among several family members or friends, the majority of care is typically provided by one individual [9].

Caregiving for individuals with dementia can be particularly burdensome due to the myriad needs and symptoms associated with the condition [10]. Dementia often begins with mild memory problems, language difficulties, and impairments with activities of daily living [11]. Behavioral symptoms can include unintentional wandering, repetitive questioning, shadowing, aggressiveness, sleep disorders, and hoarding [12, 13]. Many individuals with dementia also develop neuropsychiatric symptoms [1, 13, 14] such as depression, verbal and physical agitation, and anxiety and in the later stages can develop more severe symptoms such as delusions, hallucinations, and disinhibition [1, 15, 16]. These symptoms often become so severe in the later stages that they result in hospitalization [5, 6].

Because of these symptoms, caregiving for an individual with dementia is often seen as more stressful than caregiving for other elderly populations with serious disabilities, such as physical impairments [17]. Research has identified associations between symptoms of dementia and reduced caregiver mental health, including anger, burden, anxiety, depression, guilt, and worry [18, 19]. Physical health problems related to caregiving, such as decreased immune system functioning [20], hypertension [21], cardiovascular disease [22], and sleep problems [23] are also common. Social functioning problems include relationship challenges [24], greater family dysfunction [25], feelings of isolation, and inadequate social support [26].

Health-related quality of life (HRQOL) has been shown to be reduced in dementia caregivers [27–29]. HRQOL integrates physical health, psychological functioning, social relationships, personal beliefs, and perceptions of health-related events into a cumulative construct tapping an individual's overall well-being [30, 31]. Research has begun to investigate HRQOL in dementia caregivers from Colombia [32]. When compared to a control group, it was found that dementia caregivers in Colombia scored significantly poorer on each subscale of the SF36, indicating notably lower overall HRQOL for these individuals.

Despite the reduced mental health and HRQOL shown in dementia caregivers, many also report a variety of positive experiences related to caregiving and exhibit little distress [33]. Resilience—effective coping and adaptation when faced with loss, hardship, or adversity [34]—has been identified as a protective factor against caregiver stress [35, 36]. Similarly, optimism—a general positive outlook on life [37]—has been associated with improved dementia caregiver mental health [9, 38]. Sense of coherence is an individual's orientation towards handling stress, namely, the degree to which an individual believes that he or she can comprehend, manage, and make meaning of a challenging life event [39]. SOC has been noted in dementia caregivers, namely, related to the level of caregiver burden experienced [40].

Overall, the extant literature suggests that personal strengths are associated with improved dementia caregiver psychosocial functioning. Yet, no research has investigated whether these protective factors are associated with caregiver mental and physical HRQOL, and by extension these potential associations have not been examined among dementia caregivers in Latin America. Considering that the estimated prevalence rates of dementia in Latin America match to those in developed countries [41], a need to examine dementia care in Latin America is clear. The purpose the current study was to examine whether resilience, optimism, and sense of coherence are associated with mental and physical HRQOL in dementia caregivers from Argentina and Mexico. It is hypothesized that these personal strengths will be positively associated with both mental and physical HRQOL.

## 2. Method

### 2.1. Participants

Participants for the present study were dementia caregivers from Argentina (*n* = 110) and Mexico (*n* = 20). Participants were recruited from two sites: Centro de Enseñanza Técnica y Superior University in Baja California, Mexico, and Instituto de Neurosciencias de San Lucas in Rosario, Argentina. Caregivers were defined as individuals providing daily care for an individual diagnosed with dementia. Inclusion criteria were that caregivers had to be 18 years of age or older, identify as the primary caregiver of the person with dementia, have provided care for at least three months, be well-informed regarding the patient's medical and family history, and have no history of serious psychiatric or neurological disorders.

The majority of the caregiver sample (77.7%) was female. Most of the participants were married (76.9%); 12.3% were single; 4.6% divorced; 0.8% civil union; 0.8% separated; and 0.8% widowed. The majority of the caregivers were family caregivers, but nearly 5% of the sample included nonfamilial caregivers. Specifically, almost equal parts of the sample were the spouse (43.8%) or child (43.1%) of the individual with dementia, 7.7% aunt or uncle, 2.3% “other,” 1.5% professional caregiver, 0.8% parent, and 0.8% friend. The mean age of caregivers was 56.84 years (SD = 13.18). Participants had provided care for an average of 46.94 months (SD = 26.66), and the average weekly time spent caregiving was 68.48 hours (SD = 30.64). Caregiver level of education included incomplete primary school (1.5%), completed primary (14.6%), incomplete baccalaureate (3.1%), complete baccalaureate (37.7%), incomplete technical school (1.5%), complete technical school (3.8%), incomplete university (3.1%), complete university (30.8%), and postgraduate (3.8%). In regard to household income, 0.8% earned less than minimum wage, 50.7% one to three times minimum wage, 24.6% four to five times, and 23.8% more than five times minimum wage.

### 2.2. Measures

#### 2.2.1. Short Form Health Survey (SF-36)

A Spanish version of SF-36 [42] validated on a Colombian sample [43] was used to assess eight domains of overall HRQOL: physical functioning, social functioning, physical role-limitations, emotional role-limitations, general health, mental health, pain, and vitality. SF-36 subscale scores range from 0 to 100, with higher scores indicating greater perceived HRQOL. On the SF-36, HRQOL can be broken down into two general factors by calculating the means of mental HRQOL (e.g., vitality, social functioning, mental health, emotional role-limitations) and physical HRQOL (e.g., physical functioning, physical role-limitations, pain, general health; [42]). The instrument has demonstrated adequate internal consistency, with Cronbach's alphas generally above 0.85 [44]. The SF-36 has been used extensively in Latin America with previous research on caregivers of individuals with neurological conditions such as multiple sclerosis [45], traumatic brain injury [46], and dementia [47].

#### 2.2.2. Resilience Scale for Adults (RSA)

The RSA [48, 49] was used to assess caregiver resilience. The scale is comprised of 36 items utilizing a seven-item Likert scale with five subscales that include personal competence, social competence, family coherence, social support, and personal structure. Each subscale demonstrated adequate internal reliability (0.90, 0.83, 0.87, 0.83, and 0.67, resp.) in the original validation [49]. Example items include “I believe in my own abilities” and “Believing in myself helps me to overcome difficult times.”

#### 2.2.3. Sense of Coherence Scale (SOC)

The SOC [50], validated for Spanish speakers [51], was used to assess sense of coherence. The scale is comprised of 13 items utilizing a seven-point Likert scale and has a three-subscale structure including meaningfulness, comprehensibility, and manageability. Cronbach's alphas have ranged from 0.70 to 0.95 in 127 studies [52]. Example items included “Do you have the feeling that you really do not care about what is going on around you?” and “Do you have the feeling that you are in an unfamiliar situation and do not know what to do?”

#### 2.2.4. Life Orientation Scale-Revised (LOT-R)

The LOT-R [53] assessed caregiver dispositional optimism. The scale is comprised of 10 items utilizing a four-item Likert scale with one total score. The original instrument has demonstrated adequate internal consistency with Cronbach's alpha of .82 [53]. Example items included “In uncertain times, I usually expect the best” and “I do not get upset too easily.”

### 2.3. Procedure

Participants at Centro de Enseñanza Técnica y Superior University in Baja California, Mexico, were recruited by word of mouth, flyers, and telephone contact utilizing a list of numbers generated from the Alzheimer's Foundation of Baja California. Although three caregiver participants were administered the measures on site at the Alzheimer's Foundation, 85% of the participants were administered the measures via telephone. Participants at Instituto de Neurociencias de San Lucas in Rosario, Argentina, were recruited during routine visits to the attending neurologist. Caregivers were administered the measures during the appointment. Informed consent was obtained from all participants, and the data were collected in accordance with Institutional Review Board Approval at each facility.

### 2.4. Statistical Analyses

Two multiple regression analyses were conducted to examine which caregiver personal strengths (e.g., resilience, sense of coherence, and optimism) were associated with two aspects of HRQOL (e.g., physical HRQOL, mental HRQOL). These regressions were computed using AMOS 16.0 [54] in order to derive latent variables when possible which tend to be more accurate construct estimates than manifest variables. Because the sample size in the current study (*n* = 130) was below the sample size of 200 recommended for conducting structural equation modeling in rehabilitation research [55], estimates of fit were not reported or interpreted, as they would likely be inaccurate. Instead, standardized beta weights for the independent variables and the amount of variance explained in the dependent variables were the primary focus.

In each regression, the three personal strengths were entered simultaneously as independent variables, and the dependent variable was either physical or mental HRQOL. The latent variables in the models included resilience (made up of the five subscales of the RSA), sense of coherence (made up of the three subscales of the SOC), mental HRQOL (made up of the SF-36 subscales of Vitality, Social Functioning, Mental Health, and Role Limitations-Emotional), and physical HRQOL (made up of the SF-36 subscales of Physical Functioning, Role Limitations-Physical, Pain, and General Health). Optimism was the only manifest independent variable (comprised of the LOT-R total score). A correlation matrix of all manifest indices of the primary constructs in the current study was then computed using SPSS 21.0.

## 3. Results

### 3.1. Descriptive Statistics

The means of the manifest variables for mental HRQOL were as follows: vitality M = 55.15, SD = 12.53, range = 10.00–100.00; social functioning M = 78.65, SD = 18.87, range = 25.00–100.00; role-emotional M = 63.08, SD = 44.75, range = 00.00–100.00; and mental health M = 56.43, SD = 13.2, range = 16.00–100.00. The means of the manifest variables for physical HRQOL were as follows: general health M = 72.50, SD = 21.28, range = 15.00–100.00; bodily pain M = 88.17, SD = 18.06, range = 22.50–100.00; role-physical M = 61.63, SD = 46.88, range = 00.00–100.00; and physical functioning M = 88.15, SD = 19.25, range = 00.00–100.00.

The resilience variable means included personal competence (M = 55.47, SD = 8.20, range = 37.00–70.00), social competence (M = 40.72, SD = 5.30, range = 23.00–49.00), family coherence (M = 39.15, SD = 7.21, range = 21.00–49.00), social support (M = 48.41, SD = 5.67, range = 24.00–56.00), and personal structure (M = 24.24, SD = 2.78, range = 12.00–28.00). The means for the sense of coherence variable included meaningfulness (M = 22.83, SD = 3.76, range = 7.00–28.00), comprehensibility (M = 25.68, SD = 4.85, range = 10.00–34.00), and manageability (M = 20.61, SD = 4.22, range = 8.00–28.00). The mean for optimism was 17.26 (SD = 2.96) with a range of 7.00–24.00 and higher scores indicating greater dispositional optimism.

### 3.2. Regressions with Personal Strengths and HRQOL

**Figure 1:**
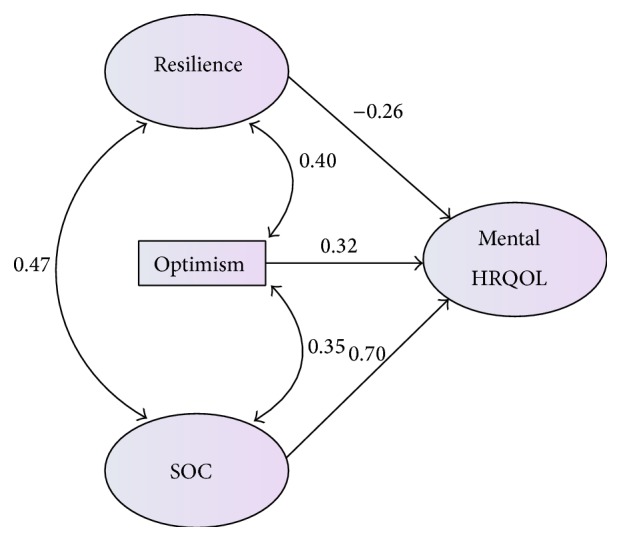
*Note*. SOC = sense of coherence; HRQOL = health related quality of life.

In the first regression (Figure 1), the personal strengths collectively accounted for 58.4% of the variance in caregiver mental HRQOL. Resilience (*β* = −.255, *P* < .039), sense of coherence (*β* = .704, *P* < .001), and optimism (*β* = .319, *P* < .002) were significantly and independently associated with caregiver mental HRQOL.

**Figure 2:**
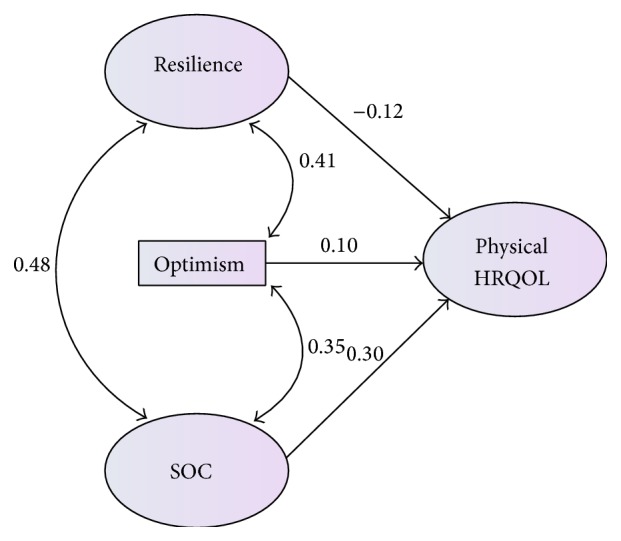
*Note*. SOC = sense of coherence; HRQOL = health related quality of life.

In the second regression (Figure 2), the personal strengths together accounted for 8.9% of the variance in caregiver physical HRQOL. Sense of coherence (*β* = .298, *P* < .028) was significantly and independently associated with caregiver physical HRQOL, while resilience (*β* = −.125, *P* = .342) and optimism (*β* = .097, *P* = .378) were not.

### 3.3. Bivariate Correlations among Personal Strengths and HRQOL

A bivariate correlation matrix was then created among all manifest indices of the primary constructs in the current study (Table 1). All personal strengths were significantly related to each other, and mental and physical HRQOL were highly correlated. All personal strengths were positively associated with mental HRQOL, but only sense of coherence was positively correlated with physical HRQOL.

## 4. Discussion

The extant literature has begun to demonstrate associations between personal strengths and enhanced psychosocial functioning of dementia caregivers [36], but these relationships have not been examined in the context of dementia caregivers in Latin America. To attend to this gap in the research, the present study examined whether resilience, optimism, and sense of coherence (SOC) were associated with mental and physical HRQOL in dementia caregivers in Mexico and Argentina. Bivariate correlations and multiple regressions generally supported the hypothesis that these personal strengths would be associated with caregiver HRQOL, although the effect was substantially stronger for mental HRQOL than physical HRQOL.

In the two multiple regressions, SOC was independently associated with both mental and physical HRQOL. According to Antonovsky's [56] salutogenic theory and empirical findings in a variety of samples focusing on the relationship between SOC and HRQOL [52], SOC may act as an internal resource augmenting control over one's life and health. Positive appraisals of the meaningfulness, comprehensibility, and manageability of dementia caregiving challenges in the current study may have acted as psychological resources to bolster healthy behaviors and increase positive affect, each related to greater mental and physical health [57, 58]. Latino caregivers often derive meaning from caregiving [59], so it is not surprising that SOC was associated with both components of HRQOL. It could be interpreted that individuals who value caregiving may also make positive appraisals of the meaningfulness of challenging events and engage in healthy behaviors surrounding those events, thus maintaining their mental and physical HRQOL. Future treatments focusing on SOC and caregiving values may be efficacious in increasing HRQOL among Latin American caregivers.

The finding that dementia caregiver resilience was independently associated with mental HRQOL is in line with previous dementia caregiver studies conducted in the United States, which have demonstrated associations between resilience and increased mental health in dementia caregivers [35, 36, 60]. Resilience is considered to act as a protective factor by increasing the quality of caregiver coping and overall adaptation to difficulties related to caregiving [34]. It could be inferred that a similar pathway to better mental health occurs in dementia caregivers in Latin America. Among other personal strengths, future intervention research would benefit from focusing on dementia caregiver resilience in Latin America in order to augment caregiver coping and mental health.

The finding that optimism was independently associated with dementia caregiver mental HRQOL also conforms to previous findings on dementia caregivers in North America [61]. The positive expectancies caregivers place on coping options and subsequent outcomes are thought to increase caregivers' perceptions of available coping techniques [62, 63]. These positive expectancies and effects on mental health are likely also present for dementia caregivers in Latin America. Cognitive behavioral interventions that improve the optimism of dementia caregivers in this region may diversify the number of coping strategies they have available and improve their mental HRQOL.

Perhaps the most notable finding was that these personal strengths accounted for over six times the variance in mental HRQOL as physical HRQOL. Despite the evidence for salutary independent associations between resilience and optimism with mental HRQOL, these independent associations did not emerge with physical HRQOL. This finding generally conforms to that from previous research in other geriatric and rehabilitation populations showing that resilience [64] and optimism [65] are associated with mental HRQOL, but not with physical HRQOL. Caregiving for family members with a health condition is valued in Latino cultures [66], and increased informal care predisposes family members to a myriad of physical caregiving burdens [17]. It is possible that positive psychological resources do not protect against the direct physical responsibilities of caregiving (e.g., patient surveillance, physical care, and lack of sleep) as strongly as they do mental HRQOL.

The findings of the present study suggest that HRQOL in dementia caregivers may benefit from interventions focusing on the role of personal strengths. There is much evidence to suggest that interventions for dementia caregivers have positive psychosocial effects for caregivers and individuals with dementia [67, 68]. Effects for caregivers include increased overall mental health and greater caregiver skill competencies, while individuals with dementia have been found to benefit through increased mental health and delayed admission for inpatient services [69].

Considering the central role of SOC in caregiver HRQOL in the present study, interventions for dementia caregivers in Latin America may benefit from strengthening caregivers' positive appraisals of the meaningfulness, comprehensibility, and manageability of caregiving activities. Previous dementia caregiver research in developed nations has demonstrated associations between dementia caregivers' HRQOL and SOC [40, 70], and a base of dementia caregiver research in Latin America has demonstrated associations between HRQOL and other important caregiving outcomes such as mental health [47].

A coping skills training program is a common component of interventions to reduce caregiver burden [68]. A variety of intervention studies have shown positive changes in SOC in diverse patient populations after meaning-focused treatments [71–73]. Clinicians may support dementia caregivers by examining caregivers' appraisals of the meaningfulness, comprehensibility, and manageability of challenging caregiving experiences and by reinforcing the importance of the psychosocial facets in caregivers' overall well-being. Developing treatments around these areas may have positive outcomes for the overall HRQOL of dementia caregivers in Latin America.

### 4.1. Limitations and Future Directions

Although the findings have implications for clinical practice in Latin America, the present study has several limitations and, as a result, directions for future research. The cross-sectional design of the study provides baseline evidence to examine the potential influence of personal strengths on caregiver HRQOL but does not allow for an examination of how these factors may influence caregiver HRQOL over time. Future studies would benefit from collecting longitudinal data in order to better demonstrate how caregiver strengths and HRQOL change over the course of caregiving. Second, specific clinical characteristics of the participants with dementia could not be gathered within the scope of the study. Clinical expressions, such as behavioral and cognitive functioning, of the participants with dementia could have provided greater information regarding caregiver stress. Specifically, the clinical features may have demonstrated moderating relations in which type and level of functioning may have held differing associations with caregiver stress. Future studies examining this topic would benefit from gathering a wider range of clinical data of the participants with dementia. Third, the study would have benefitted from examining the manner in which the personal strengths influence caregiver HRQOL. A larger sample would have allowed for more sophisticated analyses to examine possible meditational pathways from which the personal strengths and caregiver HRQOL may interact. Experimental designs would also provide evidence for causality in the relationship between personal strengths and caregiver HRQOL. Fourth, the study benefited from samples in two areas of Latin America; however, this cannot be considered representative of dementia caregivers in other regions of Latin America. Sampling from more diverse settings would provide a more accurate picture of findings from this study.

Despite these limitations, the results of the present study are notable due to the dearth of research on the personal strengths and HRQOL of dementia caregivers in Latin America. The results supported the hypothesis that personal strengths would be associated with mental and physical HRQOL in dementia caregivers from Latin America. These results underscore the need to construct and disseminate empirically supported interventions based in part on important personal strengths, such as meaning-focused treatments pertaining to SOC [71–73], for this underrepresented group. Improving the HRQOL of caregivers is expected to have a variety of positive effects, not only for the caregiver, but also for the quality of care they provide to individuals with dementia.

## Figures and Tables

**Table 1:** Correlations among personal strengths and HRQOL.

	1	2	3	4	5
(1) Physical HRQOL	—	—	—	—	—
(2) Mental HRQOL	.693^***^	—	—	—	—
(3) Resilience	.126	.224^**^	—	—	—
(4) Sense of coherence	.258^**^	.499^***^	.411^***^	—	—
(5) Optimism	.116	.283^***^	.482^***^	.317^***^	—

Note: ^*^ *P* < .05; ^**^ *P* < .01; ^***^ *P* < .001.
